# Performance Evaluation of Information Gathering from Edge Devices in a Complex of Smart Buildings

**DOI:** 10.3390/s22031002

**Published:** 2022-01-27

**Authors:** Florin Lăcătușu, Anca Daniela Ionita, Marian Lăcătușu, Adriana Olteanu

**Affiliations:** Automation and Industrial Informatics Department, University Politehnica of Bucharest, 060042 Bucharest, Romania; florinlacatusu94@gmail.com (F.L.); marianlacatusu94@gmail.com (M.L.)

**Keywords:** edge computing, Internet of Things, containerization, sensor systems, performance testing

## Abstract

The use of monitoring systems based on cloud computing has become common for smart buildings. However, the dilemma of centralization versus decentralization, in terms of gathering information and making the right decisions based on it, remains. Performance, dependent on the system design, does matter for emergency detection, where response time and loading behavior become very important. We studied several design options based on edge computing and containers for a smart building monitoring system that sends alerts to the responsible personnel when necessary. The study evaluated performance, including a qualitative analysis and load testing, for our experimental settings. From 700+ edge nodes, we obtained response times that were 30% lower for the public cloud versus the local solution. For up to 100 edge nodes, the values were better for the latter, and in between, they were rather similar. Based on an interpretation of the results, we developed recommendations for five real-world configurations, and we present the design choices adopted in our development for a complex of smart buildings.

## 1. Introduction

The transformation of people’s lives through the use of smart buildings has become a trend for residential purposes, university and corporate campuses, and commercial complexes, where it is important to focus on both socioeconomic and environmental factors that can be facilitated by smart technologies [1], including the Internet of Things (IoT) and cloud computing [2,3]. The multiple sensors embedded into built environments increase efficiency, security, and comfort [4], but also require new information models and architecture to gather relevant data. In this context, a complex of smart buildings is also exposed to a large variety of risks with regard to its administration and the protection of its residents [5]; therefore, it is necessary to deploy a multitude of sensors to detect motion, pressure, contact, temperature, smoke, light intensity, flame, carbon monoxide, moisture, and leaks [6].

Edge computing is a paradigm that is highly distinguishable in the IoT world and is very helpful for building monitoring systems. Khan et al. described the challenges of implementing edge computing solutions by analyzing various implementations [7]. Although edge computing is similar to cloud computing in some respects, it differs when the location of the resources is taken into account; the computing resources are situated in a local network, called the edge network, where services are provided correspondingly, as in the case of cloud implementation. In such an architecture, data that are processed on multiple different devices at the edge of the network are sent to the Cloud to be further analyzed and used by other applications. Another term that is used in conjunction with edge devices is fog computing. This includes the use of dedicated nodes to transform data that originate from edge devices and are sent via a local network. These nodes gather data from the sensing devices and process them; after that, data are sent for further processing in the Cloud [8]. The term “fog computing” has been primarily used by Cisco to define devices that can process data in the field.

By its nature, the edge network is designed to lower the latency period between the moments that data are acquired by the devices and when they are processed in the Cloud, by implementing processing nodes in the field, closer to the acquisition location. The implementation of an edge network can be applied in the case of warning and emergency alert systems, because data are processed on the monitored area, and local alarms can be triggered directly based on the values obtained from sensors. The implementation of a smart building network was discussed in [9], where architecture was proposed with the main goal being to obtain environmental parameters to reduce the energy footprint of the building.

The objective of our research was to design smart building architecture by combining the advantages provided by an edge network located within a building and the cloud hosting the monitoring application. Similar to [10], our main functionalities were monitoring multiple environmental parameters using different sensors, detecting abnormal situations, and sending notifications in both centralized and decentralized ways. The result was the Edge Watcher System (EWS), which was conceived for monitoring a complex of smart buildings and detecting emergency events. A critical part of our work was the selection of architecture to best fit this use case. An early version of this system, strictly dedicated to implementation in a university campus, is described in [11]. Given the existing design alternatives, our research focused on testing the performance of the EWS smart building monitoring system. This evaluation was essential for managing building risks because it provided the means to determine whether the EWS system runs within the desired parameters for various inputs, similar to real-world situations. The importance of this critical step mostly relies on the idea that every new development piece must be tested on different pre-defined scenarios where different performance metrics are monitored.

To properly evaluate performance, the tests covered multiple aspects for which different sections of the cloud application were verified. The first aspect was information gathering in the Cloud, and this was based on data sent by the edge nodes. The method applied simulated various edge node configurations that send data to EWS to be further analyzed. Another important aspect of this evaluation was the scale of the monitoring system based on sensors. With this setup, multiple building edge node configurations were simulated; larger buildings required multiple edge nodes in order to cover a larger area. The tests were defined and executed for scenarios corresponding to (1) a small apartment, (2) a house, (3) a small apartment building, (4) an office building, and (5) a complex of buildings, such as a university campus. In addition, more stress test cases, from 100 to 1000 nodes, were considered, and this was followed by a detailed analysis of the reports generated for all of these cases. Another aspect taken into consideration with the previously mentioned tests was the software architectural design. A comparison between a centralized solution hosted in a public cloud and a decentralized one where each building/complex of buildings has the monitoring system deployed on its own local datacenter was conducted. Similar tests were executed for the two architectural options, both including a container-based service, such as a Kubernetes cluster. This comparison showed how much cloud implementation affected the response times for the application, compared to when the same solution was deployed on the same network as the edge nodes. In [12], the topic of performance testing for a cloud-based solution is further detailed. Last, but not least, another aspect considered was the performance evaluation of the emergency detection algorithm used to decide whether the collected data are critical or not. For this purpose, it was important to see how fast the algorithm would run on a cloud-based container solution, compared to the same implementation deployed on a local container cluster. Another important factor was the algorithm’s performance against a high number of requests, represented by a large edge network located in a complex of smart buildings.

The contributions of this research are summarized below:

- The article addresses a timely issue—the use of smart building monitoring systems to identify emergency events and transmit notifications to the responsible personnel. It presents a qualitative analysis of design options considering two criteria: the location of the container-based services in the software architecture, and the edge network.
- For the containerized architecture, we set up testing environments for two architectural options: a centralized one, with a cluster hosted in a public cloud, and a decentralized one, with a similar cluster deployed in a local datacenter. The tests were executed for different numbers of edge nodes corresponding to real-life situations: a small apartment, a house, a small residential building, an office building, and a complex of smart buildings.
- Based on the measured performance, the article makes recommendations for choosing between the public cloud and local deployment with respect to the number of edge nodes used for building monitoring.
- We explain the design choices and several implementation details for a complex of smart buildings gathering data from numerous edge nodes (up to 1000), for which performance tests demonstrated that a cloud solution for identifying and notifying emergencies is expected to deliver better response times.

This paper continues with the presentation of related work (Section 2) followed by a qualitative analysis of architectural design options regarding container-orchestration system deployment and the edge topology (Section 3). Section 4 describes the testing plan, including the tools and technical settings used to execute the performance tests and evaluate the two architectural options based on Kubernetes. Section 5 presents the results obtained with JMeter regarding the execution success, the response time, the throughput, and the run time of the emergency detection algorithm. Then, Section 6 presents recommendations based on a comparison of the tested options and presents the final design choices with several details about the development of the EWS.

## 2. Related Work

The research areas related to our work and to the contributions presented above are edge computing, emergency management, and performance testing. For this paper, we analyzed the literature that presents edge computing for emergency detection (Section 2.1) and that related to performance testing (in Section 2.2); this included studies on the performance of edge architecture, among others.

### 2.1. Edge Computing for Emergency Detection

The implementation of alerting systems is described in [13] as being vital for every building and even more so for smart buildings. These systems also serve to provide effective evacuation of the occupants in case of an emergency. One of the most important alerting systems, typically present in a building, is a fire alarm, triggered when smoke is detected. In a smart building, such a system must communicate with the HVAC (Heating, Ventilation, and Air Conditioning) system to contain the fire. Moreover, when an alarm is triggered, the access control system must unlock all doors that are used for evacuation. In case of a fire, the use of the elevator is prohibited; thus, along with the detection of the fire, the elevator must be blocked if it is empty. This chain of events that must be triggered after the detection of a fire must also be shown on a control panel that is controlled by a trained operator. This system can fluidify the evacuation of all people from the affected building and provide vital data to the rescue personnel. Regarding emergency detection and warning topics, a study on the influence of deployment choices used in such a system for a university is presented in [14]. Additionally, related to our current concerns about smart buildings, the solution presented in [15] introduces communication between smart buildings at the level of a smart city and presents a discussion on how one can react under different circumstances.

Edge computing can be integrated into many application domains to process some of the data that come from a sensor network on the field. This approach can be easily integrated into a monitoring system, with the aim of detecting an unwanted event and limiting loss linked to its effects. Syafrudin et al. presented a warning system integrated into a manufacturing factory that can detect different defects and unwanted events that occur on the assembly line [16]. Edge computing can have multiple applications and be effective in multiple scenarios. Shuja et al. presented a way of improving resource management in the context of using computing tasks for big geo-textual data that are moving from centralized cloud platforms to distributed edge nodes [17]. A more detailed presentation on the integration, architecture, and possible use of sensor networks for smart cities is given in [18], where different implementations are presented, including performance testing and comparisons.

### 2.2. Performance Testing

Evaluating performance is very important in edge and cloud computing and even more so for the use of these systems in emergency management. It requires multiple tests of “responsiveness, reliability, throughput, interoperability, and scalability” under a given workload [19]. Haseeb-ur-rehman et al. developed a sensor cloud taxonomy that covers network, communication, data management, architecture, heterogeneity, and security aspects [20]. Energy consumption, delay time, scalability, latency, reliability, response time, and availability were the main objective functions considered.

Platforms that integrate IoT devices with cloud computing environments also need to measure performance parameters, such as the response time of sensor data acquisition and the throughput of the HTTP server [21]. For example, for the FIWARE platform, a cloud-based testbed is created to generate the load of protocols and emulate large-scale deployments of devices that send data [22], taking into account the cloud-based deployment, the performance observability, the massive load generation, and adherence to standards. The ability to support latency-sensitive applications of an edge cloud system was also analyzed in [23] from scalability and performance points of view. Li et al. used performance testing to evaluate their proposed replica creation algorithm based on the Grey–Markov chain model [24]. The testing simulated the data access situation in the edge cloud system, and the access frequency of the data block was calculated according to these data access situations and then used to determine the data heat in the proposed replica creation algorithm. Another experiment was conducted by Palade, Kazmi, and Clarke to evaluate the response time, the success rate of the deployed functions, and the throughput performance of open serverless frameworks in an edge computing environment [25]. This was achieved using a distributed load testing procedure and was orchestrated using a client desktop machine. In this system, each serverless framework uses a device to trigger HTTP requests to invoke the functions deployed on it; the tool is configured to perform 10 queries for various levels of concurrency. Liu et al. also simulated a large number of user access cases in the system in an edge computing environment [26] and proposed mechanisms to enhance the availability of the edge network resources and an auto-scaling mechanism for microservices in order to efficiently use the limited resources on the edge network. A comparative analysis based on an increasing computational workload in a network overhead for high-end cloud and edge servers is given in [27]. Scheuner and Leitner focused on a Function-as-a-Service performance evaluation in [28].

In various IoT and Wireless Sensor Network applications, the large volume of data collected from different sources [29] requires the optimization of data gathering techniques. The challenges of design and deployment are related to energy consumption, quality of service, security and privacy, adaptability, and localization. Several deployment possibilities, ranging from traditional infrastructure to platforms including container technology, a container scheduler/orchestrator, a storage, network, services, and continuous delivery, are discussed in [30].

A smart building integrates physical and computational elements to sustain an environment offering energy efficiency, comfort, and safety for its inhabitants. Various technologies related to data analytics, acquisition, storage, and visualization support its management systems. Performance awareness is key to the reduction of energy consumption, optimal building operation, timely detection, and the diagnosis of faults as they emerge, as well as providing the ability to spot various trends of decline in deteriorating components. A review of smart buildings based on the adoption of IoT is presented in [31]. Ferrández-Pastor et al. proposed a model that integrates smart services that are distributed using edge and fog computing techniques [32]. Santos et al. evaluated the performance of the IoT sensors and edge-fog components of smart building infrastructures using the utilization level, drop rate, mean response time, and flow rate metrics [33]. Continuous performance testing is also proposed in [34] as a service for smart buildings.

There are many tools available for testing performance. An example is the Apache JMeter, which was used to evaluate smart building systems in [19,35], and several other options are presented in [36]. Bryant and Marín-Pérez compared solutions to verify the load testing of HTTP servers, configure the interaction of virtual users with the site, and determine the concurrency of activities [30]. An analysis of the essential metrics provided by Apache JMeter is provided in [37], where it is recommended that the desired response time that is considered acceptable for the developers of the application is defined. This metric is related to the throughput, because the maximum capacity for the system is computed as the maximum supported throughput, concerning the maximum acceptable response time for the clients. For this study, different test cases are presented using metrics such as the ramp-up period, the number of virtual users, and the loop count (the number of calls performed by a user). Banias et al. developed a technique to generate test cases and highlight non-functional and functional testing, and metrics applied over REST APIs in order to analyze performance and functionality [38].

## 3. Analysis of Design Options for the Edge Watcher System

The Edge Watcher System is a building manager that was conceived to gather information from the building environment and the people inside with the purpose of notifying the responsible personnel when an emergency event is detected. A building administrator can create a configuration and set up the sensing devices according to the model of the building; every building has a different topology: a certain number of floors, rooms, hallways, etc. These elements are important because, in a building monitoring system, the location of a sensing device influences the detection of a possible problem on time. The monitoring application makes it possible to view the relationships between each floor and its sensing devices, configure the sensors, and inspect the recordings when critical values are notified.

The problem addressed in this paper is determining how to choose the architecture of a system such as the EWS, taking into consideration the multiple design options revolving around two main points: the location of the software components and the edge network topology. Multiple architecture possibilities were analyzed and subjected to a performance evaluation before choosing one to fulfill the requirements. The EWS uses a native cloud computing approach based on containers and also employs the edge computing paradigm. Therefore, we analyzed the options for:

(1). The architectural design of the container-orchestration system (analyzed in Section 3.1);
(2). The method of connecting sensing devices to the Cloud (analyzed in Section 3.2).

### 3.1. Options for the Containerized Architecture

The EWS services are based on containerization to automate application deployment and allow an easy configuration for various smart building models. Hence, regarding the location of the building manager services, there are two deployment possibilities: within a public Cloud, or within a local building datacenter.

**Figure 1:**
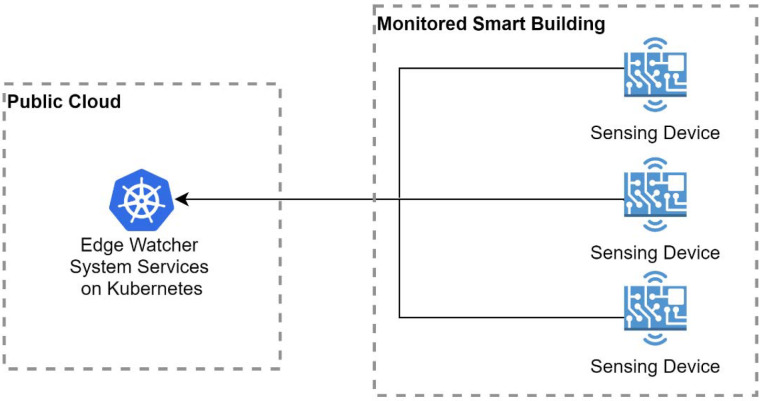
The EWS with a public cloud Kubernetes cluster.

*Architectural Option A—Public Cloud Kubernetes Cluster.* The first analyzed solution locates the EWS service on a Kubernetes cluster deployed in a public cloud. The data are collected by the cloud monitoring system directly from the sensing devices distributed throughout the building with the aim of detecting emergency situations and notifying the responsible personnel (see Figure 1). The advantage of deploying the Kubernetes cluster remotely is related to the principle of separating the monitoring system from the monitored target building. In this case, the building monitoring system would not be affected by the different outages that can appear when an emergency occurs. Nonetheless, there are other important aspects, such as maintenance costs and the initial hardware acquisition costs, which are zero with this public cloud option. There are, indeed, usage costs which, in the end, are lower compared than those required for the acquisition and maintenance of a small data center, including the personnel involved in these operations. However, a disadvantage that applies to a system using this approach is the dependence of the monitoring system on a reliable Internet connection for sending data to the cloud. This issue can be easily mitigated by providing backup connectivity in case the main Internet connectivity is not available by coupling the system with mobile Internet connectivity, which should be present on each edge node/smart sensor node, to independently send data to the monitoring system if other Internet options are not available.

In conclusion, the main advantage that supports this design option is that the monitoring system does not depend on the building resources to function, and the costs to maintain a local datacenter are removed. As a disadvantage, the Cloud datacenter is located further from the building and the requests from the sensing devices take longer to be fulfilled.

**Figure 2:**
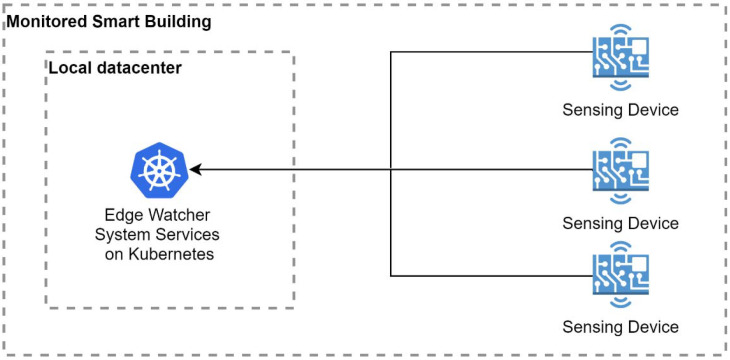
EWS with a local datacenter Kubernetes cluster.

*Architectural Option B—Local Datacenter Kubernetes Cluster.* In the second solution, the local data center design implies that the monitoring system is installed on the hardware located inside the building (Figure 2). The advantage of this approach is faster communication between the sensors and the monitoring system. Communication in the local network is faster than in the one that operates via the Internet. This is coupled with the fact that the system does not depend on having a reliable Internet connection to send environmental data to the monitoring system. The big drawback that comes with the implementation of this solution is the dependence of the monitoring system on the building’s electrical grid. When there is a problem with the electrical system, the monitoring system cannot be kept online. This issue is only applicable for the data center’s hardware. The sensing devices consisting of edge nodes and sensors are composed of low-power devices that can function on a battery for a very long time, providing the necessary data from the building environment.

Compared to the location design of the first method, with this approach, the main advantage is the lower latency period in the transmission of data between the local edge network and the local cluster. The main disadvantage is that if a critical event occurs, the monitoring system can be also affected, since it is located in the same facility.

### 3.2. Options for the Sensing Devices

The previous section analyzed design options based on the location of the monitoring system, i.e., public cloud and local datacenter deployments. Other important aspects regarding the edge topology, the options for the sensing devices, and how these devices are connected to the cloud and used to collect data for the monitoring system are discussed subsequently.

**Figure 3:**
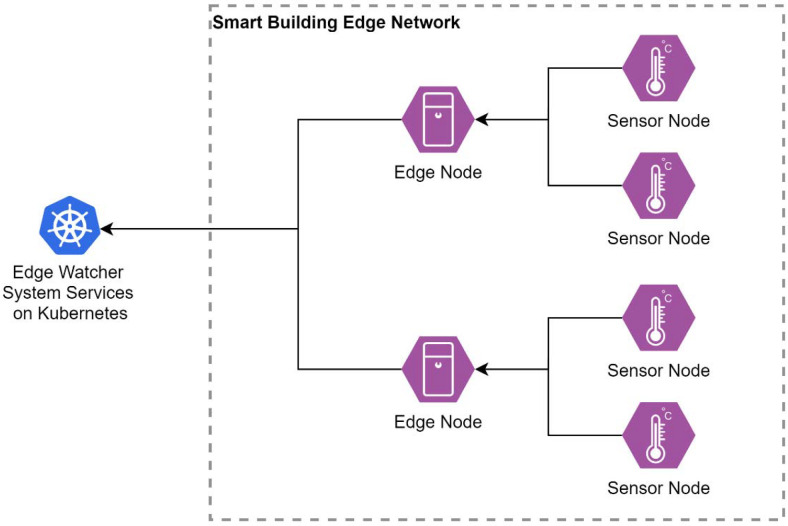
EWS design with edge nodes.

*Edge Option A—Edge Nodes.* The first design option considered for the sensing devices is based on an architecture composed of microprocessor-based edge nodes that gather data from wireless, low-power, microcontroller-based sensor nodes (Figure 3). The purpose of the edge nodes is to gather data from multiple sensing devices and send them to the cloud monitoring system. This approach can be installed in any building to monitor the environmental parameters. The edge nodes are connected to the Internet and can also function on a battery for shorter time frames compared with the sensor devices.

**Figure 4:**
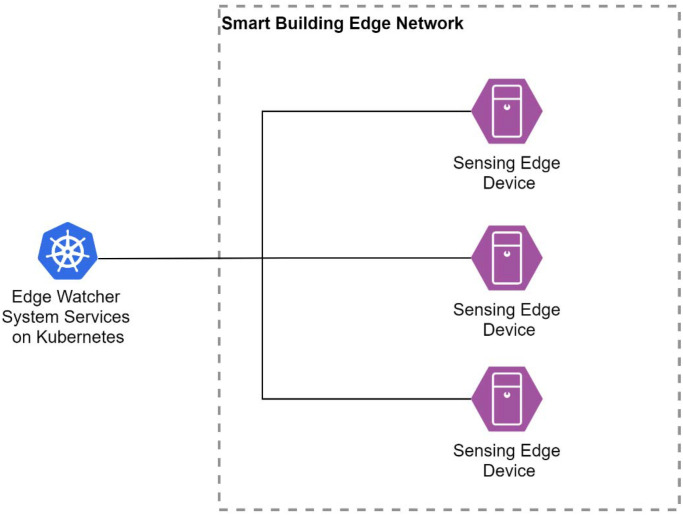
EWS design with sensing edge devices.

*Edge Option B—Sensing Edge Devices.* The second design option taken into consideration for the implementation of the sensing devices was to connect sensing edge devices directly to the cloud (Figure 4).

This system is similar to the edge node presented earlier, but the sensors are connected through a physical connection to the node. The sensing edge devices are based on microprocessors, and the number of sensors would be close to that of the microcontroller-based sensor nodes from the previous design choice. As microprocessors are more expensive than microcontrollers and consume more power, this approach would be a lot more expensive than the previous one while providing the same functionality in our use case. This is further discussed in Section 6.2.

## 4. Performance Evaluation of the Containerized Architecture Options

Based on the design options presented in Section 3.1, we hereby present the method used to determine the performance of the system with multiple sensing devices on the edge. The tests compared the performances of the two architectural design options: (A) the public cloud Kubernetes cluster; and (B) the local datacenter Kubernetes cluster. These were both connected with sensing devices through edge nodes based on the first option from Section 3.2. The tests conducted involved load and stress testing. The goal of this comparison was to gain a detailed view of the performance requirements needed for the cloud system when data are received from multiple nodes. Each test conducted corresponded to a real-world configuration for a type of building, with specific needs regarding the number of edge nodes and installed sensors required.

### 4.1. Scenarios

For both architectural options, our concern was to identify testing scenarios based on configurations of real-world building examples, from a small apartment to an entire complex of buildings, such as a university or a corporate campus. Therefore, the performance tests were executed for EWS services hosted on containerized environments following the scenarios described below. We selected examples inspired by the occupancy classification and definitions given in The International Building Code [39] and from the ten classes of buildings established in [40].

*Small Apartment.* This scenario refers to an individual unit in a residential building. We considered the setup for a small apartment with two rooms and one edge node. In this scenario, the edge node needs to collect environmental data from sensors to detect motion, temperature, and contact when the door opens or closes. For testing purposes, an API call is simulated from the edge node to the EWS, which results in the addition of a new reading in the database. The decision algorithm compares each value to a predefined threshold to verify if an alert should be considered. The scenario is equally applicable for a shop in a shopping center if the sensor monitoring is done separately by the shop tenant.

*House.* The second scenario targeted a standalone residential building. We considered the example of a detached house with five rooms and two floors. In this scenario, an edge node is installed on each floor to collect environmental data and send it to the EWS. The difference from the first scenario is in the use of two edge nodes; therefore, it may also be appropriate for a housing unit in a group of attached dwellings.

*Small residential building.* The small building scenario encapsulates a total of five simulated edge nodes installed on each floor of the building. Each node receives environmental data sent by sensors installed in the public space and inside the individual apartments situated on the same floor. The monitoring and the alert notifications are managed for the entire building by the responsible personnel.

*Office building.* In this scenario, we simulated the case of a building with 10 floors and 20 edge nodes. In this scenario, two edge nodes are installed on each floor in order to receive environmental data from the sensors and send them to the EWS for further processing. We considered this example of a non-residential building used for professional or commercial purposes, because this type of building is more often used as a smart building; therefore, it can take advantage of services for emergency detection and alerting, such as those considered in our study.

*A complex of buildings.* We considered a scenario at a larger scale, corresponding to a group of related smart buildings with residential, business, or institutional usage. They may correspond to a shopping center, a university, a corporate campus, or a residential complex. They may occupy a smaller or larger area, with multiple sensors used to measure environmental data connected to EWS through multiple edge nodes. We first considered three cases for performing load and stress testing:

- 50 edge nodes—scattered around multiple buildings that comprise the complex;
- 100 edge nodes—to test the capacity to work under a high load by registering a high number of environmental data points sent within a short period of time;
- 1000 edge nodes—to test the limits of the cluster configuration and the capacity to simulate 1000 requests sent to the system without a ramp-up period; the requests are sent immediately to the server, and it has to address each request as soon as the previous one has been fulfilled.

Then, we also executed tests for edge node numbers of between 100 and 1000 with steps of 100.

### 4.2. Performance Test Settings

For the EWS, performance testing was conducted using Apache JMeter version 5.4.1, in order to simulate the requests from the edge nodes and verify whether they were addressed without error and if the response time was low enough. The machine used to run the JMeter tests was powered by a 4-core Intel i7 CPU, coupled with 8 GB of RAM.

**Figure 5:**
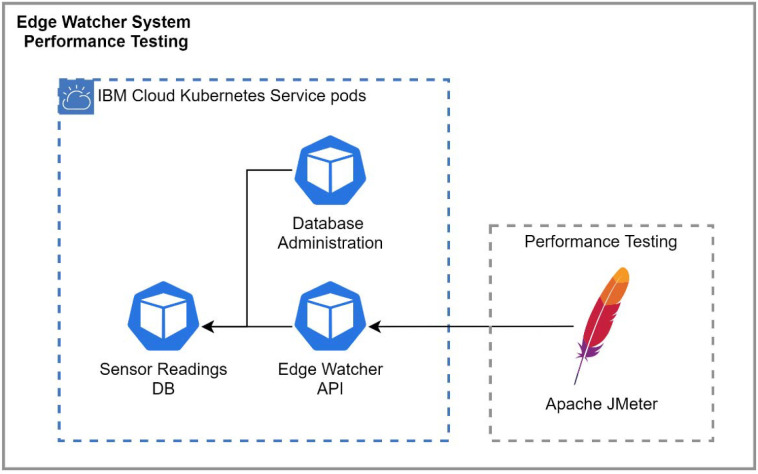
Performance testing architecture.

The performance testing architecture is illustrated in Figure 5. Test cases were developed for different sizes, starting from a small apartment and finishing with a complex of buildings. The tool setup consisted of creating threads to simulate groups of edge nodes and executing requests against the EWS reporting API. Regarding the JMeter configuration, there were three important parameters to set up:

- The number of threads: represents the number of edge nodes used to send environmental data to the application;
- Ramp-up period: the time that it would take to get to the full number of threads;
- Loop count: the number of tests to be executed.

To plan the tests conducted for the EWS, the setup had to be changed for each scenario. The ramp-up period was set to 0, the loop count was 1, and the number of threads changed with the number of desired simulated edge nodes.

The actual functionality targeted for these tests was the capacity of the application to successfully receive the environmental data from the edge nodes and insert them into the database. Therefore, a POST request had to be executed against the EWS API. The simulation of the HTTP requests for web services was based on the approach described in [41]. In the Apache JMeter interface, a dedicated HTTP Request section can be added to the test where a request can be configured [42]. The JMeter provides a dedicated setup page to configure the necessary calls to the application. Here, a POST request is configured, providing the server hostname and the port for the target API, along with the path on which the request is performed. The most important setup for this POST request is the actual body data sent. This JSON body represents the simulated data sent by the edge nodes and represents environmental readings taken from the different sensors employed throughout the monitored building. Moreover, each sensor that is connected to a node is registered to the EWS during the building setup stage, where floors, nodes, and sensors are configured for each building. In this way, one can filter the data collected by each sensor, providing separate views.

In addition to the settings presented earlier, related to the HTTP request body and path, another important setting that has to be addressed is the HTTP request header. The accept parameter indicates the response content that the Apache JMeter wants to receive from the server. For the current case, this was a JSON response. The content-type parameter indicates the request body data type. The EWS requires authentication in order to access its APIs. Therefore, the authorization token must be provided to perform calls on the API.

### 4.3. Containerized Environment Setup

To test the containerized architecture options for the EWS, we implemented two configurations, corresponding to the analysis presented in Section 3.1:

(A) The IBM Cloud Kubernetes cluster was implemented for the public cloud Kubernetes cluster design option with the following technical details:

- Deployment in IBM Cloud data centers;
- Free one node Kubernetes cluster with two cores and 4GB of RAM (default free tier);
- Kubernetes version: 1.21.7 (default).

The IBM Cloud Kubernetes service is the main offering for deploying containerized implementations in IBM Cloud targeting production workloads [43].

(B) The Docker Desktop local cluster was implemented for the local datacenter Kubernetes cluster design option with the following technical details:

- Deployed on a Windows machine with 4-core Intel i7 CPU, coupled with 8 GB of RAM-Windows Subsystem for Linux (WSL) 2;
- Docker Desktop WSL 2 backend version 4.1.1 with Kubernetes;
- Memory and CPU allocated dynamically to improve resource consumption;
- Kubernetes version 1.21.5 (default).

The Docker Desktop is a tool that is usually deployed on Windows and Mac machines. It is used to easily deploy a container development environment that contains the Docker engine and the Kubernetes container orchestration [44].

**Figure 6:**
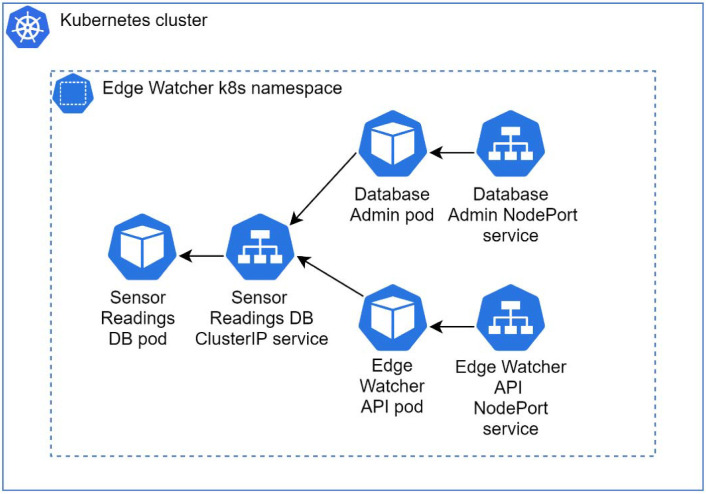
Kubernetes deployment architecture.

Both the IBM Cloud Kubernetes cluster and the Docker Desktop local cluster feature the same deployments that consist of different pods used by the application. A pod is the smallest Kubernetes unit and can contain one or more containers. A container implements the necessary software components and configurations used to run the application [45]. The Sensor Readings pod contains the MySQL database, which is used to store the data collected from the edge nodes (see Figure 6). It is exposed through a ClusterIP service that only permits network access from the local cluster network. This service is accessed by two pods. The first one is the Database Admin, which is used to configure the database after deployment and to create the necessary application users. This pod is exposed through a NodePort service that permits external access by using the worker node public IP. The Edge Watcher API pod hosts the system backend, which is also exposed by a NodePort service, in order to receive calls from different users or from the building edge nodes. In the case of a testing scenario, the Apache JMeter executes requests against the EWS API, which are processed and saved into the MySQL database.

Regarding the configuration files, there is a difference between the IBM Cloud Kubernetes cluster and the Docker Desktop local cluster. For the IBM Cloud Kubernetes service, the IBM Cloud Container registry is used in order to store the EWS Node.js Docker image. Because this is a secured private registry, the Edge Watcher Kubernetes deployment contains the imagePullSecrets attribute that points to the container registry Docker configuration secret, which contains the access token. For the Docker Desktop local cluster, we created a local Docker registry that does not require authentication and, hence, can only be accessed from the local machine. As a result, the above-mentioned attribute is not necessary for local deployment on the Docker Desktop Kubernetes.

### 4.4. Performance Metrics

The Apache JMeter makes it possible to output an HTML result that offers multiple parameters that are related to the response time of the tested request. The attributes that are relevant to our study are the execution errors, the response time, and the throughput (to determine the system’s performance). They are associated with the following metrics:

- Error % (for the execution);
- Average response time;
- Minimum response time;
- Maximum response time;
- Median response time;
- Percentiles;
- Transactions/s (for the throughput).

The ratio of the failed requests, the Error %, should be 0 for a successful test.

The Median response time metric was computed by ordering the numbers in the dataset in ascending order. Afterward, if the number of values was odd, the median was taken from the center value. If the number of values was even, the median was taken as the arithmetic average of the numbers from the center [46].

(1) $$Median\left(X\right)=\{\begin{array}{c} X\left[\frac{n+1}{2}\right],\;\;n\;is\;odd\\ \frac{X\left[\frac{n}{2}\right]+X\left[\frac{n+2}{2}\right]}{2},\;\;n\;is\;even \end{array}\;,$$

where *X* is a list of ordered numbers, and *n* represents all numbers [47].

“Percentiles” represents the percentage of values placed below the nth percentile. The rest of the values were calculated by subtracting the nth percent from 100 [46]. Among the results obtained with JMeter were the 90th, 95th, and 99th percentiles. This means that the request time for the nth percent of the user calls should fall below these numbers. This is a good indication of the application performance for most users.

“Transactions/s” was used to measure the throughput as it represents the number of transactions that an application can handle. This metric is very important because it can show the capacity of a certain website to address the needs of its respective users [48].

(2) $$\mathrm{Troughput}=\frac{\mathrm{number}\;\mathrm{of}\;\mathrm{requests}}{\mathrm{total}\;\mathrm{time}},$$

### 4.5. Emergency Detection Algorithm Used in Testing

Another factor influencing performance testing besides the JMeter HTTP calls was an algorithm that was used to verify whether the collected sensor values exceeded a pre-defined (configurable) threshold. For the performance testing of the algorithm, a Node.js dedicated library that measures the run times of different functions was employed.

**Figure 7:**
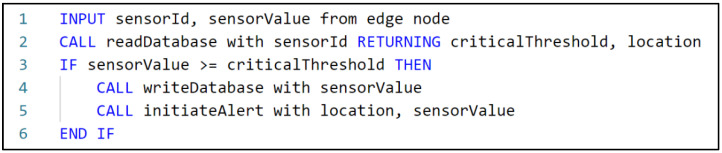
Algorithm for emergency detection.

The purpose of the algorithm, located on the containerized environment, was to filter the data received from sensors in order to detect a possible emergency (see Figure 7) and then to send alerts to the responsible personnel. Hence, the environmental data were compared to a pre-defined threshold that was set up during the configuration of the EWS for each building/complex of buildings it was applied to. The critical values were stored in the database, and alerts were sent to the responsible personnel based on these values.

For the algorithm, the metric employed was the average execution time, which was computed on the CSV file generated using the execution-time Node.js library. For each of the performance tests, there are two cases presented, corresponding to the two architectural options.

## 5. Results

For each of the two containerized environment setups (i.e., the IBM Cloud Kubernetes cluster and the Docker Desktop local cluster—see Section 4.3), multiple performance tests were executed to simulate the relevant scenarios identified in Section 4.1. The monitored results correspond to the metrics obtained for the JMeter HTTP calls, simulating the information gathered from a number of edge nodes (presented in Section 4.4) plus the run time of the decision algorithm for emergency detection, which was measured with a Node.js library, as explained in Section 4.5. Thus, the performance testing was executed both for the local and the public cloud cluster implementations.

**Figure 8:**
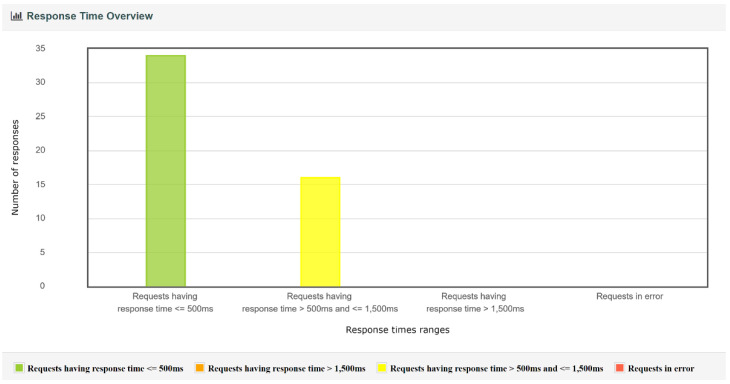
Response time overview for 50 edge nodes and the IBM Cloud.

**Figure 9:**
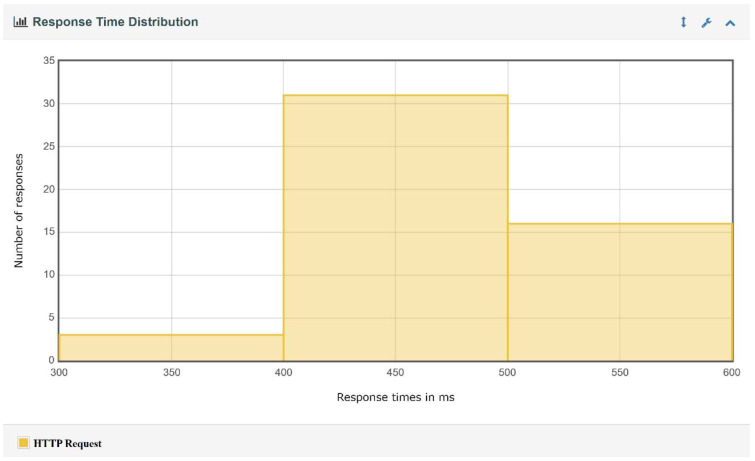
Response time distribution for 50 edge nodes and the IBM Cloud.

**Figure 10:**
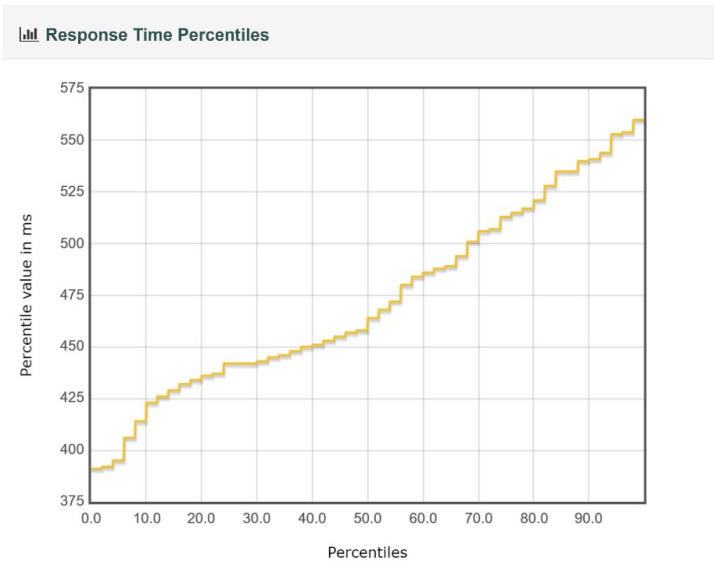
Response time percentiles for 50 edge nodes and the IBM Cloud.

**Figure 11:**
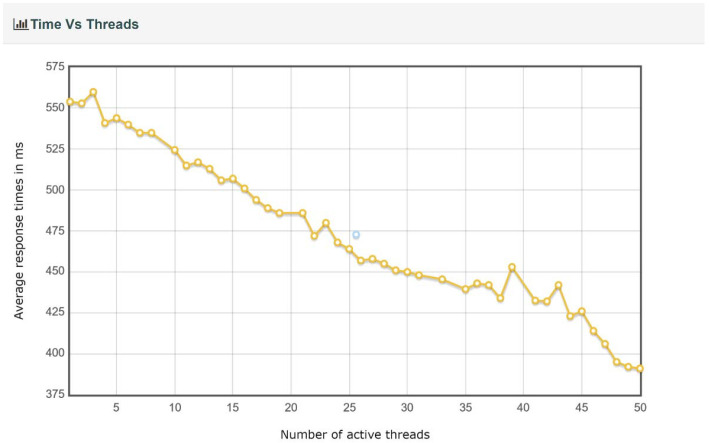
Time versus threads for 50 edge nodes and the IBM Cloud.

For each test executed, JMeter generated a report containing charts and tables regarding the throughput, the response time percentiles, the response time overview, and the response time distribution, etc. Several examples of charts obtained from JMeter for the IBM Cloud Kubernetes cluster and 50 edge nodes are presented in Figure 8, Figure 9, Figure 10 and Figure 11. Note that, in Figure 8, color-coded reference time ranges for evaluating the performance are presented, according to JMeter: green for response times of less than 500 ms, yellow for response times between 500 and 1500 ms, and orange for response times greater than 1500 ms; red is reserved for errors in requests. A more detailed distribution of the response times is given in Figure 9.

Thus, we generated 30 JMeter reports, each containing a multitude of graphics and data. To analyze them comparatively, several important results are summarized in Table 1 for the IBM Cloud Kubernetes cluster and in Table 2 for the Docker Desktop local cluster.

**Figure 12:**
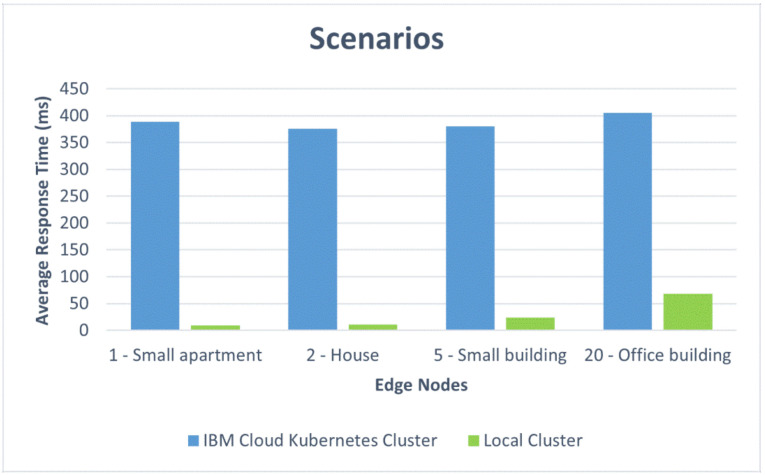
Comparative response times for different scenarios.

**Figure 13:**
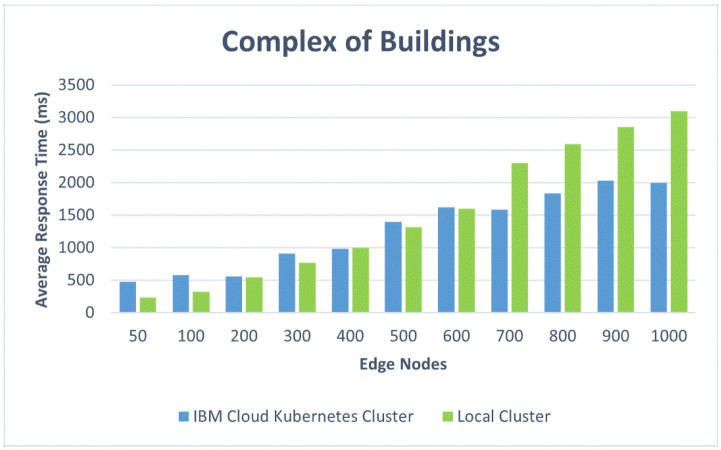
Comparative response times for the complex of buildings.

**Figure 14:**
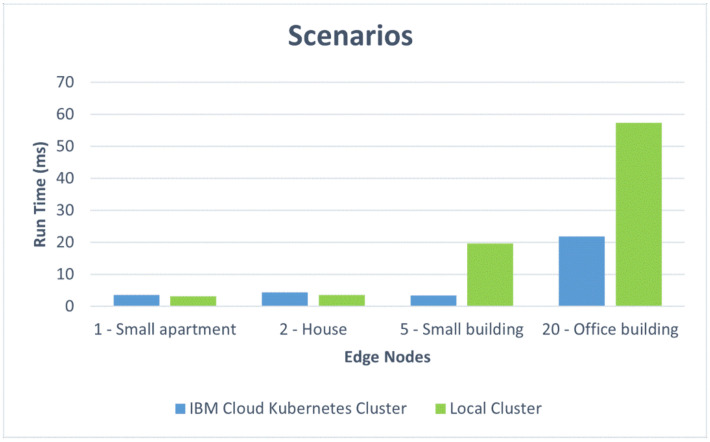
Comparative run times for different scenarios.

**Figure 15:**
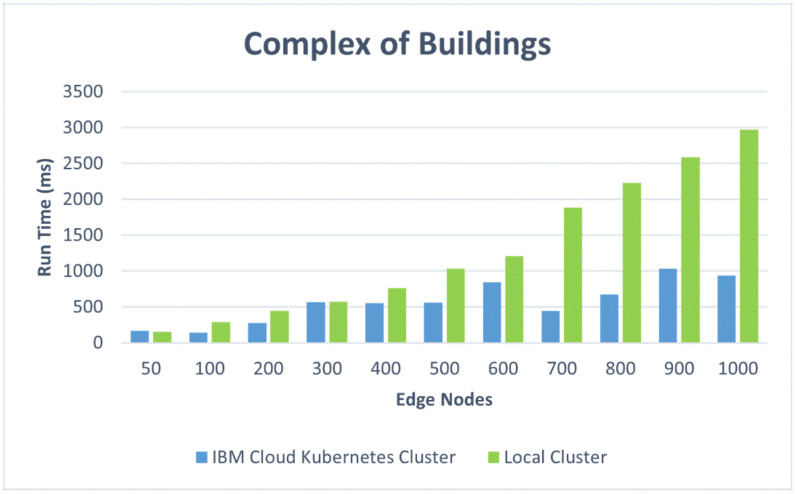
Comparative run times for the complex of buildings.

We also present the results in comparative graphics to show the difference in the average response time for the two architectural design options: the IBM Cloud Kubernetes cluster and the Docker Desktop local cluster. Figure 12 shows the response times versus the number of edge nodes corresponding to the first four scenarios: one edge node for the small apartment, two edge nodes for the house, five edge nodes for the small residential building, and 20 edge nodes for the office building. Figure 13 represents the response times for a complex of buildings versus the number of edge nodes, ranging from 50 to 1000. One can, thus, observe the influence of the container orchestration decentralization. Figure 14 and Figure 15 illustrate similar comparisons for the run time of the decision algorithm for emergency detection.

We observed that the Docker Desktop local cluster worked faster for the first four scenarios with up to 20 edge nodes as a result of its location. However, the IBM Cloud Kubernetes cluster also performed very well for the first four scenarios, keeping an average response time of under 500 ms [49].

The IBM Cloud Kubernetes cluster (corresponding to architectural option A) performed better than the Docker Desktop local cluster (architectural option B) on the most demanding test cases, from 700 to 1000 edge nodes (almost one second faster). Option A was represented by a cloud-dedicated cluster that was more powerful than Option B, which runs on a workstation. However, in cases with more than 100 edge nodes, both performed worse than the 500 ms limit, which is usually employed for web applications. As a conclusion to these tests, in a production environment, a more powerful cluster is recommended in order to provide satisfactory performance for cases with more than 100 edge nodes.

## 6. Discussion

This section discusses the performance for the five scenarios considered in our study (in Section 6.1) and the final design choices (in Section 6.2), taking into account both the testing results from Section 5 and the qualitative analysis from Section 3. This work is the basis of the Edge Watcher System’s development, for which some implementation details are given in Section 6.3.

### 6.1. Performance Comparison Based on Scenarios

This section discusses the results obtained for the implementation of the two architectural options for each of the scenarios presented in Section 4.1.

*Small apartment.* For one edge node, the differences that can be seen in the response times (Figure 12) are mainly due to the fact that the Docker Desktop local cluster is deployed on a local environment and the latency is lower compared with the IBM Cloud Kubernetes cluster setup. Even though there were large differences between the respective response times, the results for the public cloud implementation still fall under an acceptable response time, i.e., under 500 ms. The run times of the algorithm for emergency detection were similar.

*House.* For the two edge node configurations, the readings were not very different to those obtained with the 1 edge node approach (from the small apartment scenario). The results for both hosting options (Docker Desktop local cluster and IBM Cloud Kubernetes cluster) were very similar to those obtained for the previous scenario, offering a good performance.

*Small residential building.* With the increase in the number of edge nodes tested, most of the changes related to the response time were registered on the Docker Desktop local cluster (Figure 14). The most notable difference and the cause for this slower response was related to the emergency detection algorithm run time, which increased significantly in the case of the Docker Desktop local cluster. For the IBM Cloud Kubernetes cluster, the values remained similar to those obtained with the two-edge-node approach.

*Office building.* After increasing the requester number to 20 edge nodes, the response times increased for both tested approaches: the Docker Desktop local cluster and the IBM Cloud Kubernetes cluster. Nonetheless, the results show that both local and cloud approaches are able to handle an office building where 20 edge nodes send environmental data at the same time.

*A complex of buildings.* For this scenario, the size of the complex and the number of edge nodes implanted can make an important difference from a performance point of view; the cases that were simulated are discussed separately. For both implementations of the containerized environment setup, the 50-edge-node experiments showed an increase in the average response time, with a more visible change for the Docker Desktop local cluster, where a developmental virtual machine was installed on a workstation. The smaller increase in response time for the IBM Cloud Kubernetes cluster was influenced by the use of a dedicated cluster to provide stability. Figure 8 shows that two-thirds of the requests had a response time below the recommended value of 500 ms (most between 400 and 500 ms), and the other third had response times of between 500 and 575 ms (Figure 9). Figure 10 presents the exact percentiles. The average response time was higher when the number of active threads was low (Figure 11), because there were only a few users waiting for their calls to be executed; the others had already been served, i.e., the load was high. The run time of the algorithm for emergency detection increased for both approaches, providing a similar value. For the 100-edge-node stress test, both setups showed an increase in the average response times (Figure 13). For the IBM Cloud Kubernetes cluster, the run time of the algorithm for emergency detection for the cloud system was very similar to the result obtained for the 50-edge-node experiment. For the Docker Desktop local cluster, the run time increased (Figure 15), again illustrating the advantage provided by a dedicated cluster compared to the local workstation. Between 200 and 600 nodes, the response times for the two design options were quite similar; yet, starting from 700 edge nodes, a clear advantage towards the IBM Cloud Kubernetes cluster was shown, due to the fact that both the response times and the run times were better. The 1000-edge-node experiment verified the behavior of the system when 1000 calls were sent to it. The error rate for each of the systems was 0, which indicates that all environment data were successfully added to the EWS database. A big difference that only occurred for this experiment, but not the other scenarios, is that the Docker Desktop local cluster provided a higher average response time compared with the IBM Cloud Kubernetes cluster. For the other scenarios, the result was the opposite; the reason for this is that the request was sent locally, and the time difference was accounted for by the fact that, for the public cloud option, the request was sent via the Internet. Regarding the algorithm for emergency detection, the Docker Desktop local cluster implementation provided a run time that was almost three times higher than that obtained with the the IBM Cloud Kubernetes cluster.

This underlines the fact that the use of a dedicated cloud cluster can offer a more consistent performance under a high load. However, as a general remark for the results related to the public cloud option, an average response time of almost 2000 ms is too high for this type of system. Based on these results, for a complex of smart buildings, a more powerful cluster configuration than the one tested in this study and described in Section 4.3 is recommended. The 1000 edge nodes represent a load experiment that may occur in real-world situations for large building complexes, such as smart campuses or large shopping centers.

An important limitation when working with a cloud solution may be related to the location of the datacenter. This choice influences the network latency present in the call to the application. For the tests presented in the paper, the free Kubernetes cluster was automatically deployed in the IBM Cloud Dallas datacenter using the IBM Cloud Console, which does not allow for a change in this location. However, for the purpose of our evaluation, we also tested the network latency for different IBM Cloud datacenters using SoftLayer, an official tool available for the IBM Cloud; it provides the ability to test the network latency from a public IP to an IBM Cloud datacenter using the ping command. Thus, the Round-Trip Time (RTT) can be measured for packets sent from the source to the destination, including the time passed until confirmation is sent back to source. We considered three different locations for the IBM Cloud datacenter, Dallas, Oslo, and Milan, and obtained the following RTT average values: 156, 62, and 44 ms, respectively. The location of the testing machine was Bucharest. Therefore, for a production IBM Cloud Kubernetes cluster, Milan would be the best datacenter to install EWS for monitoring a building in our area. On average, the RTT was found to be approximately 110 ms lower than in Dallas, providing an improvement in the response time with 20% from the limit value.

To summarize, for a small apartment with one edge node, the response times were better for the Docker Desktop local cluster, but they also had good values for the public cloud Kubernetes cluster, whereas the run times for the decision algorithm were similar for the two architectural options. For a house with two edge nodes, the results were quite similar to those from the previous scenario. For a small residential building with five edge nodes, the situation was not very different in terms of the average response times, but there was a significant increase in the run times for the Docker Desktop local cluster. For an office building with 20 edge nodes, the response times and the run times increased with respect to the previous scenarios for both architectural options, but they remained within acceptable limits. In conclusion, for situations with less than 50 edge nodes (corresponding to a single building in our simulations), the test results showed an average response time below the typical limit accepted in JMeter for web applications for both design options with an advantage given to the local datacenter Kubernetes cluster, due to its shorter response times. However, for a complex of smart buildings, especially for one with more than 600 edge nodes, the results prove the superiority of the architectural option based on a public cloud Kubernetes cluster, with the recommendation being to use a more powerful cluster configuration for very large complexes that need up to 1000 nodes. These results were obtained for a free default cluster with one worker node in IBM Cloud and for the Docker Desktop local cluster installed on a workstation. A production-level cluster or a high-performance local datacenter would improve the performance of the EWS under high loads, as well as increasing the cost.

### 6.2. Final Design Choices

In Section 3, we analyzed several design options for the EWS, considering two criteria: the containerized architecture and the edge network topology. After the qualitative evaluation, the method applied for the performance testing of the two architectural design options, (A) the public cloud Kubernetes cluster and (B) the local datacenter Kubernetes cluster, was described in Section 4. Further, this section presents the design choices made for developing EWS.

*Containerized Architectural Choice*: Based on the test results from Section 5 and the performance comparison for the five scenarios from Section 6.1, we conclude that architectural choice A (public cloud Kubernetes cluster) provides a stronger performance for a load corresponding to a complex of smart buildings, where the number of edge nodes used for gathering information is large. Another reason for this choice is the separation of the building monitoring system from the actual monitored building. The main advantage in case of an emergency that could produce an outage within the building is that the system would not be affected, and it would retain the information in regard to the event. Another major reason for this choice is related to the initial and maintenance costs, which are lower with the public cloud approach. Cloud services also have a guaranteed Service Level Agreement that assures the system can run for more than 99.9 percent of the time. This aspect is crucial for a building monitoring system that includes the detection of different emergencies that can occur in the building in its scope.

*Edge Choice*: Based on the qualitative analysis presented Section 4, we are in favor of the design choice that uses sensor nodes connected to microprocessor-powered edge nodes and not sensing devices based on microprocessors that are directly connected to the EWS services on Kubernetes. The edge node’s role is to gather data, perform some basic decentralized processing, e.g., for detecting when to activate local alarm devices, and sends data to the EWS to be processed in a centralized way using algorithms that require more resources. To estimate the costs corresponding to the two options for the edge network, we assumed that the same types and number of sensors were used, and we omitted other costs, such as those related to the containerized architecture. Let us consider a microprocessor-based node run with Raspberry Pi [50]—a very popular development board with the ability to act both as a sensing device (Edge Option B) and as a broker and data processor edge node (Edge Option A). For both options, Raspberry Pi provides enough computing power to run even more complex algorithms in the future [51]. The average cost for a Raspberry Pi 3 (4× ARM CPU, 1.2 GHz, 1 GB RAM, 10/100 Ethernet, 2.4 GHz 802.11n wireless) is $35. For Edge Option A, a microcontroller-based sensor node may be run with NodeMCU v3 (32-bit CPU, 80 MHz, 128 KB RAM), a microcontroller-based board with integrated Wi-Fi capability, to send data to an edge node. The average cost for a NodeMCU board is approximately $7. Thus, our estimations show that a solution containing only microprocessor-based sensing devices (Edge Option B) would cost five times more than one that also includes microcontroller-based sensor nodes (Edge Option A). This is especially important for the case of monitoring a complex of buildings with a large number of edge nodes. Therefore, Edge Option A, containing edge nodes, was selected because, overall, it is less expensive than the other option, and because it requires a smaller number of edge nodes, which is a premise for providing a better performance.

### 6.3. Edge Watcher System

Based on the results from the performance testing, in the previous subsection we concluded that our final solution for EWS should be composed of two parts: public cloud monitoring software (Architectural Option A) and sensor nodes connected to edge nodes (Edge Option A). Therefore, our system should be capable of gathering information from the edge network situated within the complex of buildings, processing it in the cloud, and deciding when to send alerts to the responsible personnel, e.g., police, building administrators, firefighters, etc. In this subsection, we present the EWS architecture and several implementation details.

**Figure 16:**
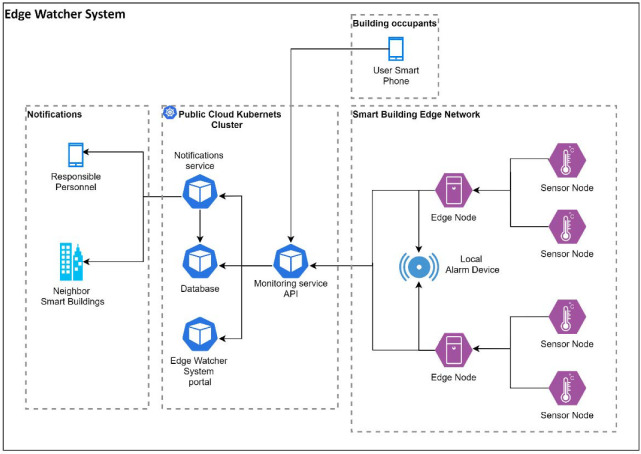
Edge Watcher System architecture.

The cloud monitoring software was run on an IBM Cloud Kubernetes cluster and represented a microservices container implementation. Multiple services comprise this system (see Figure 16), such as the Monitoring Service, which gathers information from sensors and user reports. The Notification Service is responsible for sending alerts to the responsible persons and to neighboring smart buildings from the complex that are monitored with the same EWS. The system is configurable from the EWS Portal, supporting the possibility of adding multiple buildings, edge nodes, sensors, and users. The Database represents the persistent part of the monitoring system where all configuration and data are added.

The EWS software has four main components: (1) a Web frontend written using the Angular framework; (2) a Node.js backend (Monitoring service API); (3) a MySQL database to retain the user settings, configuration sensor, and human input; and (4) the Notification service. All of these are implemented on containers, and they run on Kubernetes. As a result, there is a dedicated pod for each of these components. This application is flexible and can run in every cloud with minimal modifications.

The sensor network architecture is based on the edge computing paradigm, which assumes that supplementary data processing will be done by field devices that form an edge network. An edge node can also activate local alarm devices based on a basic comparison of the measured values to a pre-defined threshold. This can ensure a quick response in case of an emergency event before the more complex centralized alert is activated, sending notifications to the responsible personnel. The current implementation contains two edge nodes that correspond to the second scenario for a house, but the system supports the attachment of supplementary edge nodes, as they are needed for scenarios similar to those considered in our study. A building administrator can create the edge node configurations with multiple types of sensors/sensor nodes. For this use case, as well as for monitoring, the cloud system employs a dedicated web portal that is able to receive requests from the edge nodes and provide configuration files that are used at the first edge node setup. The web portal also offers visualization capabilities for the reported data.

Every building in the complex has a different structure in terms of, for example, the numbers of floors, rooms, and hallways. These factors are important because the location of a sensing device is essential for detecting a possible emergency. The EWS provides the ability to configure the parameters for each building. One can configure the edge nodes using two approaches: a dedicated menu that presents a tree-like view of the relationships between a floor and its nodes, and an offering that edits the nodes for each floor from the corresponding menu. A clear view of the nodes of the entire building architecture can be displayed. From each of these nodes, sensors can not only be configured but also inspected to see if critical data have been recorded—a feature that also appears on the main Dashboard.

## 7. Conclusions

This study evaluated several design options for a system that monitors one or multiple smart buildings with the purpose of gathering information from a large variety of sensors, detecting abnormal situations (such as flames, toxic gas, leaks, etc.), and notifying the responsible personnel when emergency events occur. The design options took into account the container-based software architecture and the edge sensing devices. In addition to a qualitative analysis, the paper presented work based on containerized environments to test the performance, which has important weight in alerting systems. The provided response times must remain under a certain threshold in order for the solution to be approved and implemented in a production environment. We implemented two containerized environment setups (an IBM Cloud Kubernetes cluster and a Docker Desktop local cluster), and we simulated the behavior for five scenarios corresponding to real-world configurations with 1 to 1000 edge nodes. For settings corresponding to a small apartment, a house, a small residential building, and an office building, the average response time was 250 ms higher for the public cloud than for the local cluster. However, for a complex of buildings with more than 600 edge nodes, the response time was 700 ms lower for the cloud than for the local solution

We used the performance evaluation findings and the edge options analysis to make design choices for the Edge Watcher System, a solution with microcontroller-based sensor nodes, microprocessor-based edge nodes, and monitoring, configuration, and notification services with an IBM Cloud Kubernetes cluster.

In terms of future research related to building monitoring for emergency detection, it is also necessary to study the dependability of such an architecture and to know how reliable it would be in a production environment. In this regard, we plan to investigate the importance of the edge nodes’ location and to implement endurance tests to provide a clear view of how the system behaves for a longer period.

## Figures and Tables

**Table 1:** Summary of the test results for the IBM Cloud Kubernetes cluster.

Test	Executions		Response Time (ms)					Throughput	Emergency Detection Algorithm
Scenario	Samples (Edge Nodes)	Error (%)	Average	Min	Max	Median	95th Percentile	Transactions /s	Run time (ms)
Small apartment	1	0	389	389	389	389	389	2.57	3.52
House	2	0	375.5	371	380	375.5	380	5.26	4.42
Small residential building	5	0	380	371	385	382	385	12.89	3.49
Office building	20	0	405.75	374	437	405.5	436.8	43.8	21.8
A complex of buildings	50	0	472.84	391	560	461	553.45	81.04	167.43
	100	0	574	376	748	574	729	115.74	144.42
	200	0	556.83	375	815	563.50	789.00	203.87	278.9
	300	0	906.49	446	1336	947.50	1309.95	196.34	564.4
	400	0	981.00	380	1461	936.00	1412.85	236.27	554.4
	500	0	1393.26	420	2090	1394.50	2014.85	212.04	560.3
	600	0	1614.98	391	2282	1602.00	2225.95	223.46	846.57
	700	0	1584.66	374	2373	1620.00	2288.90	246.05	448.3
	800	0	1832.57	398	2759	1855.0	2651.95	250.16	677
	900	0	2026.60	499	3056	1994.0	2954.85	230.00	1036.5
	1000	0	1992	384	3538	1927	3359	189.9	939.89

**Table 2:** Summary of test results for the Docker Desktop local cluster.

Test	Executions		Response Time (ms)					Throughput	Emergency Detection Algorithm
Scenario	Samples (Edge Nodes)	Error (%)	Average	Min	Max	Median	95th Percentile	Transactions /s	Run time (ms)
Small apartment	1	0	9	9	9	9	9	111.11	3.22
House	2	0	11	9	13	11	13	153.85	3.59
Small residential building	5	0	24	16	32	23	32	151.52	19.62
Office building	20	0	68.2	31	109	69.5	107.7	176.99	57.3
A complex of buildings	50	0	228.44	97	288	239	285.8	130.89	155.5
	100	0	322.33	78	460	311	447.9	175.75	288.86
	200	0	543.18	138	978.6	553.7	930.7	176.3	447.3
	300	0	768.05	147	1390	769.3	1322.1	179.2	574.5
	400	0	995.9	164	1821.6	1002.5	1741.1	179.92	762.5
	500	0	1316	181	2378.3	1318.3	2293.3	177.93	1030.9
	600	0	1597.8	360	2855.6	1515.6	2737.8	175.79	1211.2
	700	0	2296.3	426	3262.4	2094.1	3151.5	164.04	1881.5
	800	0	2589.2	363	3698.7	2515.5	3560.75	173.05	2232.5
	900	0	2849.8	147	3994.4	2842.3	3757.9	162.14	2588.6
	1000	0	3098	243	4431	2914	4284	171.59	2975.23

## Data Availability

Not applicable.
